# Engineered External Guide Sequences Are Highly Effective in Inhibiting Gene Expression and Replication of Hepatitis B Virus in Cultured Cells

**DOI:** 10.1371/journal.pone.0065268

**Published:** 2013-06-12

**Authors:** Zhigang Zhang, Gia-Phong Vu, Hao Gong, Chuan Xia, Yuan-Chuan Chen, Fenyong Liu, Jianguo Wu, Sangwei Lu

**Affiliations:** 1 State Key Laboratory of Virology, College of Life Sciences, Wuhan University, Wuhan, Hubei, China; 2 Program in Comparative Biochemistry, University of California, Berkeley, California, United States of America; 3 School of Public Health, University of California, Berkeley, California, United States of America; Drexel University College of Medicine, United States of America

## Abstract

External guide sequences (EGSs) are RNA molecules that consist of a sequence complementary to a target mRNA and recruit intracellular ribonuclease P (RNase P), a tRNA processing enzyme, for specific degradation of the target mRNA. We have previously used an *in vitro* selection procedure to generate EGS variants that efficiently induce human RNase P to cleave a target mRNA *in vitro*. In this study, we constructed EGSs from a variant to target the overlapping region of the S mRNA, pre-S/L mRNA, and pregenomic RNA (pgRNA) of hepatitis B virus (HBV), which are essential for viral replication and infection. The EGS variant was about 50-fold more efficient in inducing human RNase P to cleave the mRNA *in vitro* than the EGS derived from a natural tRNA. Following *Salmonella* -mediated gene delivery, the EGSs were expressed in cultured HBV-carrying cells. A reduction of about 97% and 75% in the level of HBV RNAs and proteins and an inhibition of about 6,000- and 130-fold in the levels of capsid-associated HBV DNA were observed in cells treated with *Salmonella* vectors carrying the expression cassette for the variant and the tRNA-derived EGS, respectively. Our study provides direct evidence that the EGS variant is more effective in blocking HBV gene expression and DNA replication than the tRNA-derived EGS. Furthermore, these results demonstrate the feasibility of developing *Salmonella* -mediated gene delivery of highly active EGS RNA variants as a novel approach for gene-targeting applications such as anti-HBV therapy.

## Introduction

**Figure 1:**
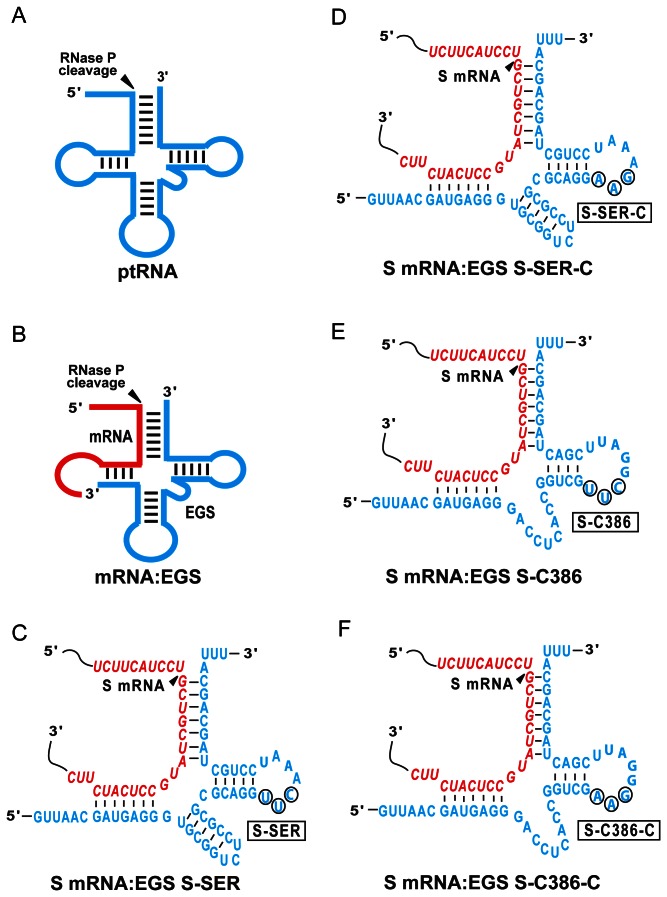
Substrates for RNase P. (A) A natural substrate (ptRNA). (B) A hybridized complex of a target RNA (e.g. mRNA) and an EGS resembling the structure of a tRNA. (C, D, E, and F) Complexes between HBV S mRNA (S RNA) sequence and EGS S-SER, S-SER-C, S-C386, and S-C386-C, respectively. The sequences of S-SER and S-SER-C that were equivalent to the T-stem and loop, and variable region of a tRNA molecule were derived from tRNA^Ser^, while those of S-C386 and S-C386-C were from EGS variant C386. Only the exact sequence of the S mRNA around the targeting site is shown (in red) and the EGS sequence is shown in blue color. The site of cleavage by RNase P is marked with an arrowhead.

Nucleic acid-based gene interfering technologies, such as antisense oligonucleotides and RNA interference (RNAi), have been shown to be a promising gene targeting approach for use in basic research and clinical therapeutic applications [1]. Ribonuclease P (RNase P) has been found in all organisms examined and its enzymatic activity is responsible for the maturation of 5’ termini of all tRNAs which account for about 2% of total cellular RNA [2–4]. This enzyme is a ribonucleoprotein complex and catalyzes a hydrolysis reaction to remove the leader sequence of precursor tRNA (Figure 1A) [2–4]. Studies on RNase P substrate recognition revealed that the enzyme recognizes the structure rather than the primary nucleotide sequence of the substrates, and can cleave a model substrate that contains a structure equivalent to the acceptor stem, the T-stem, the 3' CCA sequence, and the 5' leader sequence of a ptRNA molecule (Figure 1A) [5]. Altman and colleagues proposed that RNase P can be recruited to cleave any mRNA using a custom-designed external guide sequence (EGS) that hybridizes with the target mRNA to form a structure resembling a tRNA substrate (Figure 1B–D) [6,7]. EGS RNAs derived from natural tRNA sequences can be effective in blocking gene expression in bacteria and in mammalian cells [7–10]. EGSs have been shown to inhibit HIV gene expression and replication in human cultured cells [11]. Furthermore, we have shown that EGSs that were derived from a natural tRNA effectively induced human RNase P to cleave the mRNAs of herpes simplex virus 1 (HSV-1) and human cytomegalovirus (HCMV) *in vitro* [10,12,13]. A reduction of ~75% in HSV and HCMV gene expression was observed in viral infected cells that expressed these functional EGS RNAs.

Increasing the *in vitro* efficiency of the EGS-induced RNase P cleavage as well as its efficacy in vivo is required in order to develop EGSs for practical use both as a research tool and as a therapeutic agent for gene-targeting applications. Using an *in vitro* selection procedure, we have isolated novel EGS variants that direct RNase P to cleave HSV-1 thymidine kinase (TK) mRNA *in vitro* more efficiently than those derived from a natural tRNA sequence [12]. Little is currently known about how these EGS RNA variants increase their activity in directing RNase P to cleave a target mRNA.

Hepatitis B virus (HBV), which chronically infects more than 400 million individuals worldwide, is a leading viral cause of liver disease including viral hepatitis and hepatocellular carcinoma (HCC) [14–16]. While a preventive vaccine is available, developing new compounds and novel strategies to inhibit HBV replication in hepatocytes is central for treating HBV-positive individuals due to the side effects of the current FDA-approved therapies and the emergence of drug-resistant HBV strains [15]. For example, small interfering RNAs (siRNAs) and EGSs have been constructed to induce RISC RNase and RNase P, respectively, to cleave target HBV mRNAs and inhibit HBV gene expression in vitro and in vivo [17–21]. Compared to the RNAi approach, which induces the cellular RISC RNase to cleave target mRNAs [1], the EGS-based technology is unique in inducing endogenous RNase P to cleave particular RNAs. Furthermore, the RNase P-mediated cleavage is specific and does not generate nonspecific “irrelevant cleavage” that is observed in RNase H-mediated cleavage induced by conventional antisense phosphothioate molecules [3,22]. Thus, EGSs may present promising gene targeting agents for therapy of human diseases including HBV infection [20,21].

To develop EGSs for anti-HBV therapy in vivo, one of the most important issues is the delivery of these agents to hepatocytes in the liver. Attenuated strains of invasive bacteria such as *Salmonella* have been shown to function as a carrier system for delivery of nucleic acid-based vaccines and anti-tumor short hairpin RNAs (shRNAs) [23–26]. In these studies, attenuated *Salmonella* was introduced with plasmid constructs containing the transgenes under the control of an eukaryotic expression promoter. The bacteria was then used to target specific cells such as macrophages and epithelial cells, leading to transgene expression [24]. *Salmonella* may represent unique delivery agents for gene therapy as they can be administrated orally and can target specific tissues and cells. The liver represents the major *in vivo* reservoir for *Salmonella* following their systemic dissemination, and hepatocytes can be invaded and infected by *Salmonella* . Thus, these cells are considered an optimal target for a *Salmonella* -based gene target therapy [24,25].

In the present study, an EGS variant was used to target the overlapping region (S RNA) of the S mRNA, pre-S/L mRNA, and pregenomic RNA (pgRNA) of HBV, which are essential for HBV replication and infection [15]. We investigated the activity of the constructed EGSs in inducing RNase P to cleave the target HBV S RNA and its efficacy in inhibiting HBV gene expression and replication in cultured cells. The EGS variant, S-C386, was about 50 fold more active in directing RNase P to cleave the target S RNA than S-SER, the EGS derived from a natural tRNA sequence. Following *Salmonella* -mediated gene delivery in cultured HBV-carrying cells, S-C386 was more effective in inhibiting viral gene expression and DNA genome synthesis than S-SER. A reduction of about 97% in the level of HBV RNAs and proteins and an inhibition of about 6,000 fold in the levels of capsid-associated HBV DNA were observed in cells that expressed S-C386. In contrast, a reduction of less than 10% in viral gene expression and DNA replication was observed in cells that either did not express an EGS or expressed EGSs that contained point mutations abolishing their ability to induce RNase P-mediated cleavage. Our results provide direct evidence that engineered EGS RNAs are highly effective in blocking HBV gene expression and DNA replication. These results also demonstrate the potential of generating highly active EGS variants and using them for anti-HBV therapy. 

## Materials and Methods

### Mapping of the accessible regions of viral mRNA in human cells

Human hepatoma HepG2 (American Type Culture Collection, Manassas, VA) and HepG2.2.15 cells were maintained in Dulbecco’s modified Eagle medium (DMEM) supplemented with 10% heat-inactivated fetal calf serum as described previously [27,28]. Hep2.2.15 cells were derived from HepG2 and contain a full length HBV DNA genome. HepG2.2.15 cells were seeded for 8–24 h prior to the treatment with dimethyl sulphate (DMS). Cells were incubated with 5 mL of fresh media that contain 1% of DMS for 5–10 min [29,30], and then washed 3 times with phosphate-buffered saline (PBS) that contained 1 mM β –mercaptoethanol, and finally lysed by adding cell lysis buffer (150 mM NaCl, 10 mM Tris–HCl pH 7.4, 1.5 mM MgCl_2_, 0.2% NP40) [29,30]. Total RNAs were isolated from the lysates by phenol-chloroform extraction, followed by ethanol precipitation. To carry out primer extension assays to identify the DMS modification sites, oligonucleotide primers of 20 nt complementary to several regions of the targeted RNA were synthesized chemically and 5’-labeled by T4 polynucleotide kinase in the presence of γ-[^32^P]-ATP. Primer extension experiments were carried out by incubation of 8 µL of a mixture of total cellular RNA (10 µg) and 50,000 cpm of primers, 4 µL 5× RT buffer, 2 µL 10 mM dATP, 2 µL 10 mM dTTP, 2 µL 10 mM dGTP, 2 µL 10 mM dCTP, 0.5 µL RNasin, and 1 µL AMV reverse transcriptase (Promega, Inc., Madison, WI). After incubation at 42°C for 2 h, the reaction products were extracted with phenol chloroform, then precipitated by cold ethanol, and finally separated in 8% denaturing gels. The sites that blocked the primer extension reaction by reverse transcription, which were revealed by analyzing the gels using a STORM840 Phosphorimager, represented the potential locations modified by DMS [29,30].

### Construction of EGS RNAs and RNA substrate for studies *in vitro*

The DNA sequence that encodes HBV substrate s38 was constructed by PCR using pGEM3zf(+) as a template and oligonucleotides AF25 (5'-GGAATTCTAATACGACTCACTATAG-3') and sS38 (5’-CGGGATCCATCCAGCGATAGCCAGGACAAGTTGGAGGCCTATAGTGAGTCGT ATTA-3') as 5' and 3' primers, respectively.

The DNA sequences coding for the EGSs were synthesized by the polymerase chain reaction (PCR), using construct pTK112 DNA or pC386 as the templates [10,12], and were cloned under the control of the T7 RNA polymerase promoter. To construct pS-SER, the 5' and 3' primer were oligoS-SER-5 (5’-GGAATTCTAATACGACTCACTATAGGTTAACGAUGAGGGTGCGGTCTCCGCGC-3’) and oligoS-SER-3 (5'-AAGCTTTAAATGCTGCTAGCAGGATTTGAACCTGCGCGCGGAGACCGCAC-3'), respectively. To construct pS-SER-C, the 5' and 3' primer were oligoS-SER-5 and the oligoS-SER-C-3 (5'-AAGCTTTAAATGCTGCTAGCAGGATTTCTTCCTGCGCGCGGAGACCGCAC-3'), respectively. To construct pS-C386, the 5' and 3' primer were oligoS-C386-5 (5’-GGAATTCTAATACGACTCACTATAGGTTAACGAUGAGGGACCTCACCG-3’) and oligoS-C386-3 (5'-AAGCTTTAAATGCTGCTAGTCGAATCCGAACGACCGGTGAGGTC-3'), respectively. To construct pS-C386-C, the 5' and 3' primer were oligoS-C386-5 and oligoS-C386-C-3 (5'-AAGCTTTAAATGCTGCTAGTCGAATCCCTTCGACCGGTGAGGTC-3'), respectively.

### RNase P assay and *in vitro* studies

We performed the assays for the binding between EGSs and S mRNA sequence *in vitro* as described previously [10,31,32]. In brief, various concentrations of EGS (0.0005–50 nM) were preincubated in buffer B (50 mM Tris, pH 7.5, 100 mM NH_4_Cl, 10 mM MgCl_2_, 3% glycerol, 0.1% xylene cyanol, 0.1% bromphenol blue) for 10 min before mixing with an equal volume of 1–10 pM substrate RNA preheated under identical conditions. The samples were incubated for 10–120 min to allow binding, then loaded on a 5% polyacrylamide gel, and run at 10 watts. The electrophoresis running buffer contained 100 mM Tris-Hepes, pH 7.5, and 10 mM MgCl_2_ [31]. The value of K_d_ was then extrapolated from a graph plotting percentage of product bound *versus* EGS concentration. The values were the averages of five independent experiments [12].

Human RNase P was prepared from HeLa cellular extracts as described previously [10,32]. We incubated the EGSs and [^32^P]-labeled s38 with human RNase P at 37^°^C in buffer A (50 mM Tris, pH 7.4, 100 mM NH_4_Cl, and 10 mM MgCl_2_). Cleavage products were separated in denaturing gels and analyzed with a STORM840 phosphorimager (GE Healthcare). Assays to determine kinetic parameters were performed under multiple turnover conditions, as described previously [12,33]. We withdrew aliquots from reaction mixtures at regular intervals and analyzed the cleavage products in polyacrylamide-urea gels. The values of K_m(apparent)_ and V_max(apparent)_ were obtained from Lineweaver-Burk double-reciprocal plots [12,33,34], and the values were the average of five experiments in triplicate.

### Analysis of *in vitro* growth kinetics of *Salmonella*

*Salmonella* strain SL201 was derived from the auxotrophic *Salmonella* *typhimurium* *aroA* strain SL7207 (a gift from Bruce A. D. Stocker (Stanford University, CA, USA)) [35] by deleting the coding sequence of *msbB*, following the experimental procedure as described previously [36,37]. To analyze the growth of *Salmonella* in LB broth, a single colony was cultured in 2 ml LB broth at 37°C with shaking at 250 RPM overnight (> 15 hours) [37]. Fifty microliters of the culture were then inoculated into 5 ml fresh LB broth and cultured at 37°C and 250 RPM. At 0, 2, 4, 6, 8, 10, 12, 14, 16, and 24 hours after inoculation, the bacterial culture was collected and used for analysis by limiting dilution in 96-well plates, and then plated on LB agar plates to determine their CFU/ml. Each sample was analyzed in triplicate and the analysis was repeated at least three times. The average value of CFU/ml was used to generate the growth curve [37].

### Expression of EGS RNAs by *Salmonella* -mediated delivery in cultured cells

*Salmonella* carrying different EGS constructs were generated by transforming strain SL201 with plasmid pU6, pU6-S-SER, pU6-S-SER-C, pU6-S-C386, pU6-S-C386-C, or pU6-TK112. Construct pU6 contained the GFP expression cassette and the small U6 RNA promoter used for the expression of EGS RNAs in mammalian cells. In gene transfer experiments to express EGS RNAs, we infected cells (1x10^6^ cells) (pre-treated with IFN-γ (150 U/ml) (R&D Systems Inc., Minneapolis, MN) for at least 12 hours) with *Salmonella* at a multiplicity of infection (MOI) of 10-20 bacteria/cell. To allow phagocytosis to occur, we incubated the cultures at 37^0^C for 30 minutes. We then added to the cultures with fresh medium containing gentamicin (20 µg/ml) and incubated them for the indicated time periods before harvesting cells.

To assay the expression of the EGSs, total RNAs were isolated from cells using Trizol (Invitrogen, San Diego, CA) and digested with DNase I to remove the genomic DNA, as described previously [7,10,12,27]. The RNA samples were then separated in a 2.5% agarose gel that contained formaldehyde, transferred to a nitrocellulose membrane, hybridized with the [^32^P]-radiolabeled DNA probes that contained the DNA sequences coding for S-C386 and S-SER, and finally analyzed with a STORM840 phosphorimager (GE Healthcare) [10]. The radiolabeled DNA probes used to detect EGS RNAs were synthesized from constructs pS-C386 and pS-SER with a random primed labeling kit (Roche Applied Science).

The cytotoxicity of the EGS expression in combination with *Salmonella* infection was assessed by an MTT assay (Sigma). Cells grown in 96-well plates were infected with SL201 carrying empty vector construct pU6 and EGS-containing constructs (i.e. pU6-S-SER, pU6-S-SER-C, pU6-S-C386, pU6-S-C386-C, or pU6-TK112). At different time points, 3-(4,5-Dimethylthiazol-2-yl)-2,5-diphenyl tetrazolium bromide (MTT; Sigma) (5 mg/mL in PBS) was added to each well, and cell viability was determined following the manufacture’s recommendation. The absorbance was measured at 570 nm on a microplate reader. All experiments were performed in four wells and repeated three times. Furthermore, the morphology of the cells at different time points was examined using a Nikon TE300 microscope.

### Assays for HBV RNA and protein expression

We isolated the RNA and protein samples following the previously described procedures [7,10,12,27]. Proteins in cultured cells and media were assayed by enzyme-linked immunosorbent assay (ELISA) using the S antigen or E antigen diagnostic kits (Abbott Laboratories, Abbott Park, IL; Shanghai KeHua Biotech Co., Shanghai, China), following the protocols provided by the manufactures [27]. In Northern blot analysis, total RNAs were isolated from cells using Trizol (Invitrogen, San Diego, CA) and treated with DNase I to remove the genomic DNA [12,27,32]. The RNA samples were separated in 1% agarose gels that contained 6.5% formaldehyde, transferred to nitrocellulose membranes, hybridized with the [^32^P]-radiolabeled DNA probes that contained the HBV DNA sequence and human H1 RNA, and analyzed with a phosphorimager. The DNA probes used to detect HBV RNAs and human H1 RNA were synthesized from plasmids pHBV1.3 and pH1 RNA, respectively [27]. Quantitation was performed in the linear range of RNA detection.

### Assays for HBV DNA levels

To isolate capsid-associated viral DNA, we lysed the cells in buffer E (50 mM Tris, pH 7.5, 0.5% NP-40, 1 mM EDTA, 100 mM NaCl), followed by incubation with DNase I at 37°C for 6 hours [27]. We then precipitated viral cores by adding 0.5 M EDTA and 35% polyethylene glycol, followed by centrifugation. We resuspended the pellet in buffer F (10 mM Tris, 100 mM NaCl, 1 mM EDTA, 1% SDS, and 2.5 mg/ml proteinase K), followed by proteinase K digestion for 16 hours. Similarly, we first digested cultured media in lysis buffer E with DNase I for 6 hours, and isolated the encapsidated DNA by proteinase K digestion in buffer F for 16 hours. The released viral DNAs were purified with phenol and chloroform extraction, followed by isopropanol precipitation [27].

Quantitative PCR (qPCR) was used to determine the levels of capsid-associated DNA, and was carried out in an ABI 7500 device (Applied Biosystems Inc., Foster City, CA) or an iCycler Real-Time PCR Detection System (Bio-Rad, Hercules, CA). The set of TaqMan real-time PCR primers used in the analysis included 5’ primer P1 (5’-AGAAACAACACATAGCGCCTCAT-3’), 3’ primer P2 (5’-TGCCCCATGCTGTAGATCTTG-3’), and probe P3 (5’-TGTGGGTCACCATATTCTTGGG-3’) [27]. We carried out the PCR reaction as follows: 1 cycle at 50°C for 2 min, 1 cycle at 95°C for 10 min, and 40 cycles at 95°C for 15 s and 60°C for 60 s. Plasmid pHBV1.3 was diluted over a range of 10^7^ to 10^0^ and used as a standard [27]. We analyzed all samples in triplicate and repeated all analyses three times. The results were the arithmetic average of triplicate experiments.

## Results

### *In vitro* RNase P-mediated cleavage of target HBV RNA sequence by engineered EGSs.

 The S mRNA overlaps and is completely within the viral pregenomic RNA (pg RNA) and pre-S/L mRNA [15]. Both the S and pre-S/L mRNAs encode viral surface antigens (e.g. HBsAg) while HBV pgRNA serves both as the template for viral DNA genome synthesis and as the mRNA for the viral polymerase and core protein [15]. Thus, targeting the overlapping region of these transcripts should simultaneously reduce the expression of viral essential proteins and the level of viral genomic DNA, and may yield a more effective inhibition of viral replication.

Because most mRNA species inside cells are usually associated with proteins and are present in a highly organized and folded conformation, it is critical to choose a targeting region that is accessible to binding of EGSs in order to achieve efficient targeting. Previous studies in our and other laboratories have shown that in vivo mapping with dimethyl sulphate (DMS) can be used to determine the accessibility of mRNA and structure of RNAs in cells [29,30,38]. To map the regions of the S mRNA that may be accessible to DMS modification, we incubated human HepG2.2.15 cells, which carry a HBV DNA genome [27,28], with culture media that contained DMS. DMS entered the cells and modified the nucleotides of the mRNA regions that were accessible. We isolated total mRNAs from these cells, and those regions of the S mRNA that were modified by DMS were mapped by primer extension assays in the presence of reverse transcriptase. We chose a position, 260 nucleotides downstream from the translational initiation codon as the cleavage site for the EGSs. This site appeared to be one of the regions most accessible to DMS modification (data not shown). Moreover, its flanking sequence exhibited several sequence features that need to be present in order to interact with an EGS and RNase P to achieve efficient cleavage. These features include (1) the nucleotides 3’ and 5’ adjacent to the site of cleavage to be a guanosine and a pyrimidine, respectively; and (2) a uracil to be localized at 8 nt downstream from the cleavage site [7,33]. The interactions of these sequence elements with the EGS facilitate the formation of the mRNA–EGS complex into a tRNA-like structure while those interactions with RNase P are critical for recognition and cleavage by the enzyme [33].

We have previously employed an *in vitro* selection procedure to isolate EGS RNA variants that are more efficient in directing human RNase P for cleavage of the HSV-1 TK mRNA sequence than the EGS derived from a natural tRNA sequence [12]. The objective of the study was to generate active EGS variants that can be used to target an mRNA, and to study the variants to understand the mechanism of how EGS RNAs efficiently direct RNase P for cleavage of an mRNA substrate. However, little is currently known about how some of these active EGS variants increase their activity in directing RNase P-mediated cleavage *in vitro*. Variant C386 was chosen for this study because the EGS RNAs derived from this variant are among the most active EGSs in inducing RNase P to cleave the HBV S mRNA as well as the TK mRNA sequences *in vitro* (see below, Table 1). EGS S-C386 was constructed by covalently linking the EGS domain of C386 to the targeting sequences that are complementary to the S mRNA (Figure 1E). Another EGS, S-SER, which was derived from the natural tRNA^Ser^ sequence, was also constructed in a similar way and included in the study (Figure 1C).

**Table 1:** Kinetic parameters [V_max(apparent)_, K_m(apparent)_, and V_max(apparent)_ /K_m(apparent)_] in the RNase P-mediated cleavage reactions of ptRNA^Ser^ or HBV s38 RNA substrate in the presence of different EGSs.

Substrate			K_m_(µM)			V_max_ (_apparent_)(pmol·min^-1^)			V_max(apparent)_/K_m(apparent)_(pmol·µM^-1^·min^-1^)			K_d_(µM)		
ptRNA^Ser^			0.020±0.075			0.061±0.025			3.0±0.6					
S RNA (s38)														
+S-SER			0.60±0.15			0.027±0.011			0.045±0.015			1.8±0.6		
+S-SER-C			ND			ND			<0.001			1.9±0.7		
+S-C386			0.30±0.10			0.75±0.30			2.5±0.4			0.025±0.005		
+S-C386-C			ND			ND			<0.001			0.026±0.005		

Multiple-turnover kinetic analyses to determine the values of V_max(apparent)_ and K_m(apparent)_ were carried out in buffer A (50 mM Tris, pH 7.4, 100 mM NH_4_Cl, and 10 mM MgCl_2_) at 37°C, as described previously [12,33,34]. To determine the binding affinity (K_d_) between substrate s38 and EGSs, binding assays were carried out in the absence of human RNase P in buffer B (50 mM Tris, pH 7.5, 100 mM NH_4_Cl, 10 mM MgCl_2_, 3% glycerol, 0.1% xylene cyanol, 0.1% bromophenol blue), using a protocol modified from Pyle et al [31]. The values shown are the arithmetic means of five experiments performed in triplicate. ND: not determined.

**Figure 2:**
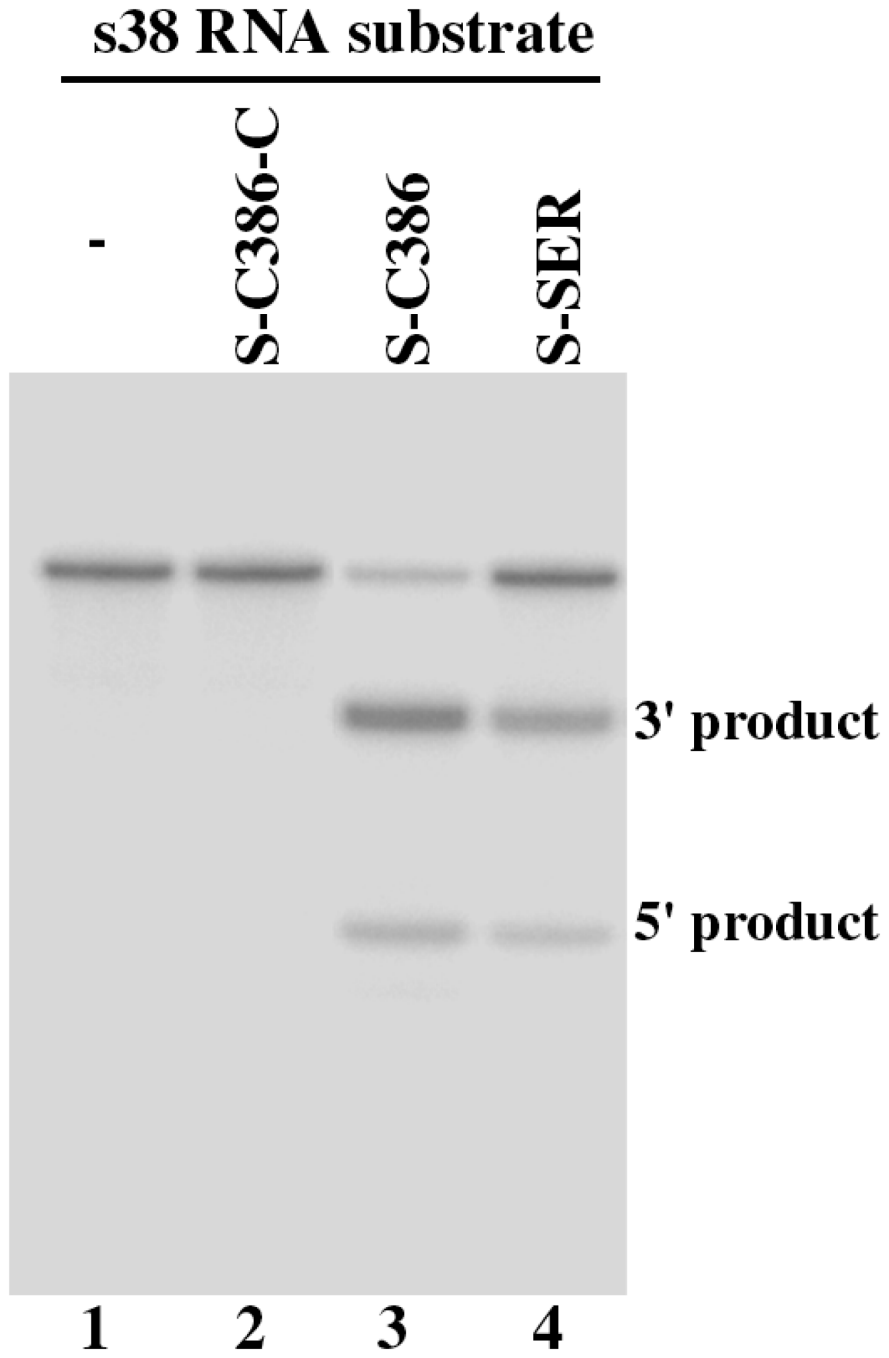
Cleavage of HBV S mRNA sequence (substrate s38) by human RNase P in the presence of different EGSs. No EGS was added to the reaction mixture in lane 1. EGS S-C386-C (10 nM) (lane 2), S-C386 (2 nM) (lane 3), and S-SER (10 nM) (lane 4) were incubated with [^32^P]-labeled S RNA substrate (20 nM) and human RNase P (2 units) at 37°C in a volume of 10 µl for 45 minutes in buffer A (50 mM Tris, pH 7.4, 100 mM NH_4_Cl, and 10 mM MgCl_2_). Experimental details can be found in Materials and Methods.

In the presence of S-SER and S-C386, we observed RNase P-mediated cleavage of substrate s38, which contained a HBV S mRNA sequence of 38 nucleotides (Figure 2, lanes 3-4). In contrast, we observed no cleavage of s38 by RNase P in the absence of these EGSs (Figure 2, lane 1). Using kinetic analyses, we determined the values of K_m(apparent)_ and V_max(apparent)_ as well as the overall cleavage efficiency [V_max(apparent)_ /K_m(apparent)_] for the cleavage reactions. S-C386 was highly efficient in directing human RNase P to cleave s38 and was at least 50-fold more efficient than S-SER (Table 1).

It is conceivable that an increase in the cleavage rate of RNase P may be due to additional tertiary interactions that potentially stabilize the mRNA-EGS complex. If this is the case, the binding affinity of the EGS variant (i.e. S-C386) to the target S RNA sequence may be better than that of the EGS (i.e. S-SER) derived from the natural tRNA sequence. The binding affinities of EGS S-C386 and S-SER to substrate s38 were determined by measuring the dissociation constant (K_d_) using gel-shift assays for separating substrate-EGS complexes in non-denaturing polyacrylamide gels. S-C386 exhibited about 70 times higher binding affinity to s38 than S-SER (Table 1). Both S-C386 and S-SER have the same antisense sequences to s38 (Figure 1C and 1E). Thus, these results suggest that the increased binding affinity and the stability of the substrate-EGS complex in the presence of S-C386 may be due to the additional tertiary interactions introduced by this EGS.

Three control EGSs were also used in our study. S-C386-C and S-SER-C were derived from S-C386 and S-SER, respectively, and contained base substitutions (5’-UUC-3’ -> AAG) at the three highly conserved positions in the T-loop of these EGSs (Figure 1D and F). The nucleotides in these three positions are highly conserved among tRNA molecules and are important for folding of the tRNA molecules and their recognition by RNase P [3], thus mutations in these positions inactivate EGS activity. Indeed, we hardly detected the cleavage of s38 by human RNase P in the presence of these two control EGSs (Figure 2, lanes 2, data not shown) and the cleavage in the presence of these two control EGSs was at least 2x10^3^-fold slower than that in the presence of S-C386 (Table 1). S-C386-C and S-SER-C contained the same antisense sequence to the target S RNA sequence as S-C386 and S-SER (Figure 1C–F), and exhibited similar binding affinities to s38 as S-C386 and S-SER, respectively (Table 1). Therefore, S-C386-C and S-SER-C can be used as controls for the antisense effect of these EGSs. EGS TK112, which targeted the HSV-1 TK mRNA [10], was also included in the study. This control EGS was used to determine if EGS with an incorrect guide sequence could affect the level of the target mRNA. We observed no RNase P-mediated cleavage of s38 in the presence of TK112 *in vitro* (data not shown).

### *Salmonella* -mediated delivery for EGS expression in cultured cells

We cloned DNA sequences encoding S-SER and S-C386 into expression vector pU6, which contains the small nuclear U6 RNA promoter for expressing EGS and a green fluorescence protein (GFP) expression cassette. The pU6-EGS constructs were transformed into *Salmonella* strain SL201 for gene delivery studies. SL201 was derived from auxotrophic strain SL7207 [35] and in addition, contained a deletion of the *msbB* gene. SL7207 has been shown to function efficiently as a gene delivery carrier for the expression of several transgenes in mammalian cells [25,26]. The *msbB* gene encodes an enzyme important for the biosynthesis of the lipopolysaccharide (LPS), which is a major virulence and proinflammatory factor expressed on the surface of *Salmonella* [39]. Deletion of *msbB* is expected to further reduce the virulence/toxicity of *Salmonella* and facilitate intracellular lysis of bacteria and release of the transgene construct, leading to efficient expression of the delivered gene in target cells.

**Figure 3:**
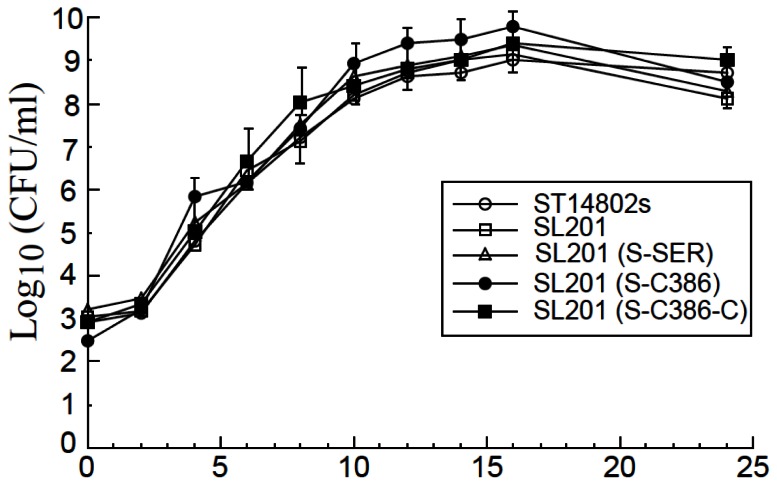
Analysis of growth in LB broth of *Salmonella* . *Salmonella* used in the study include the wild type strain ST14028s, vector strain SL201, and its derivatives that carry constructs pU6-S-SER, pU6-S-SER-C, pU6-S-C386, and pU6-S-C386-C. Experimental details can be found in Materials and Methods.

**Figure 4:**
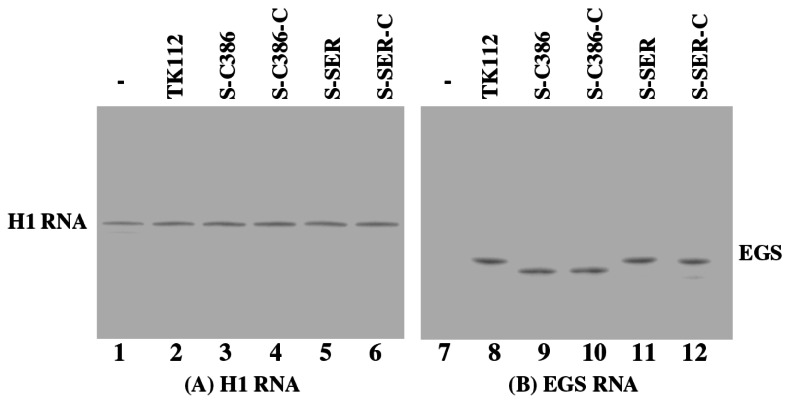
Northern analyses of the EGS RNA expression in HepG2.2.15 cells. Cells were treated with strain SL201 carrying the empty vector pU6 (-, lanes 1 and 7), pU6-TK112 (lanes 2 and 8), pU6-S-C386 (lanes 3 and 9), pU6-C386-C (lanes 4 and 10), pU6-S-SER (lanes 5 and 11), and pU6-S-SER-C (lanes 6 and 12). RNA samples (25 µg) were separated on 2% agarose gels that contained formaldehyde, transferred to nitrocellulose membranes, and hybridized to a [^32^P]-radiolabeled probe that contained the DNA sequence coding for H1 RNA (A) or EGS S-SER/S-C386 (B).

The presence of the EGS sequence cassette itself did not affect the viability of the bacterial carrier as no difference was observed in the growth kinetics of *Salmonella* carrying no constructs or various pU6-EGS constructs in LB broth (Figure 3). When human hepatoma HepG2 cells were infected with *Salmonella* carrying pU6-EGS constructs, more than 60% of cells were GFP positive at 24 hours postinfection, demonstrating efficient gene transfer mediated by *Salmonella* . The EGS expression in these cells was detected by Northern blot analysis, using human H1 RNA as the loading control (Figure 4). Similar levels of EGS RNAs were observed when cells were treated with SL201 carrying different pU6-EGS constructs at an identical multiplicity of infection (MOI) (Figure 4). Our MTT assays revealed that cells infected with *Salmonella* carrying the empty vector construct pU6 and the EGS-containing constructs were indistinguishable in terms of their growth and viability for up to three weeks (data not shown), suggesting that the expression of the EGSs did not appear to exhibit significant cytotoxicity.

### *Salmonella* -mediated gene delivery of EGS for inhibition of HBV infection and replication in cells

**Figure 5:**
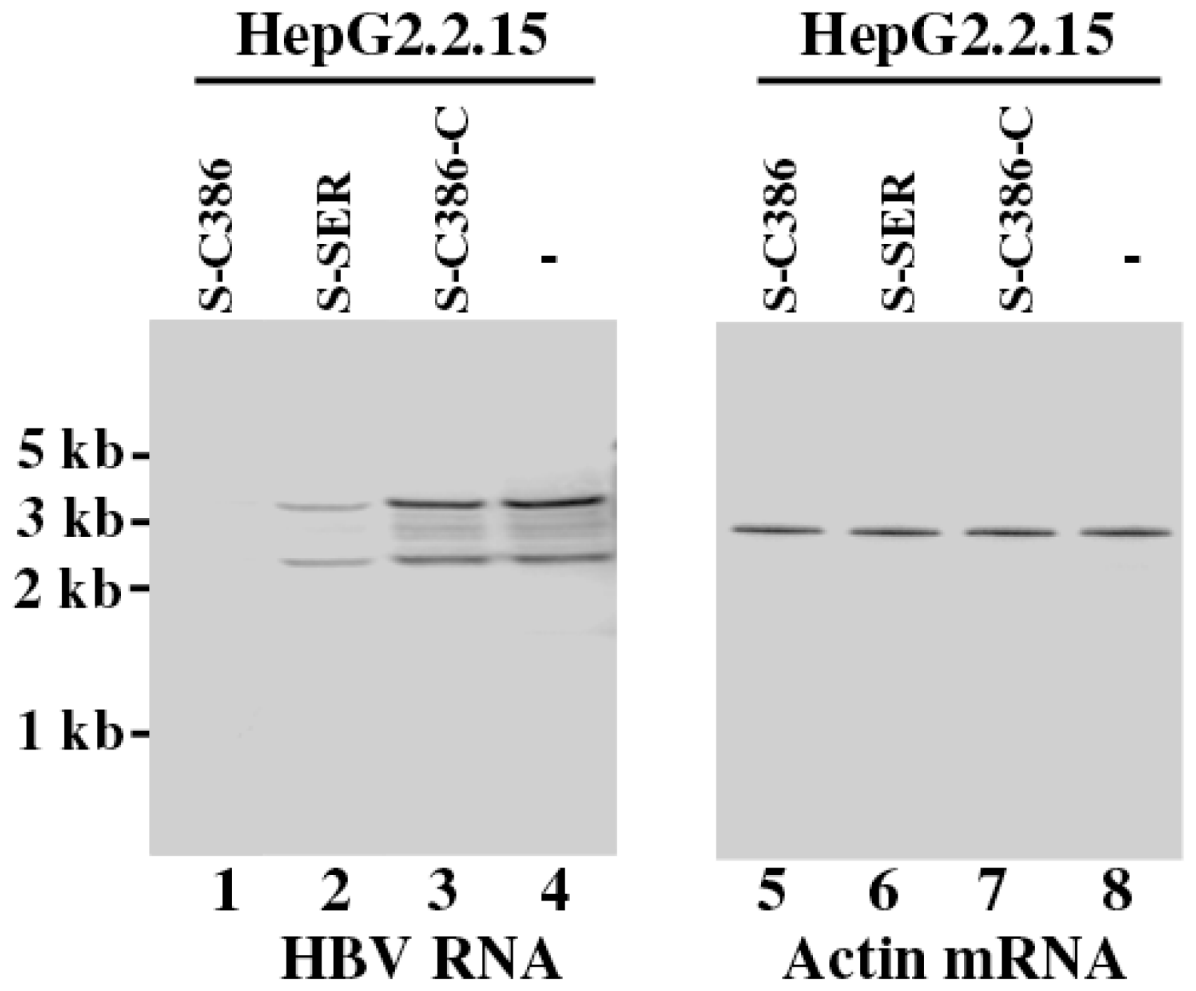
Expression levels of HBV transcripts (lanes 1-4) and loading control actin mRNA (lanes 5-8). HepG2.2.15 cells were first treated with *Salmonella* carrying pU6 (-, lanes 4 and 8), pU6-S-C386 (lanes 1 and 5), pU6-S-SER (lanes 2 and 6), and pU6-S-C386-C (lanes 3 and 7). The cells were harvested at 48 hours post treatment. RNA samples (20 µg) were separated on agarose gels, transferred to nitrocellulose membranes, and hybridized to [^32^P]-radiolabeled probes that contained the sequence of the HBV S and actin mRNAs.

**Figure 6:**
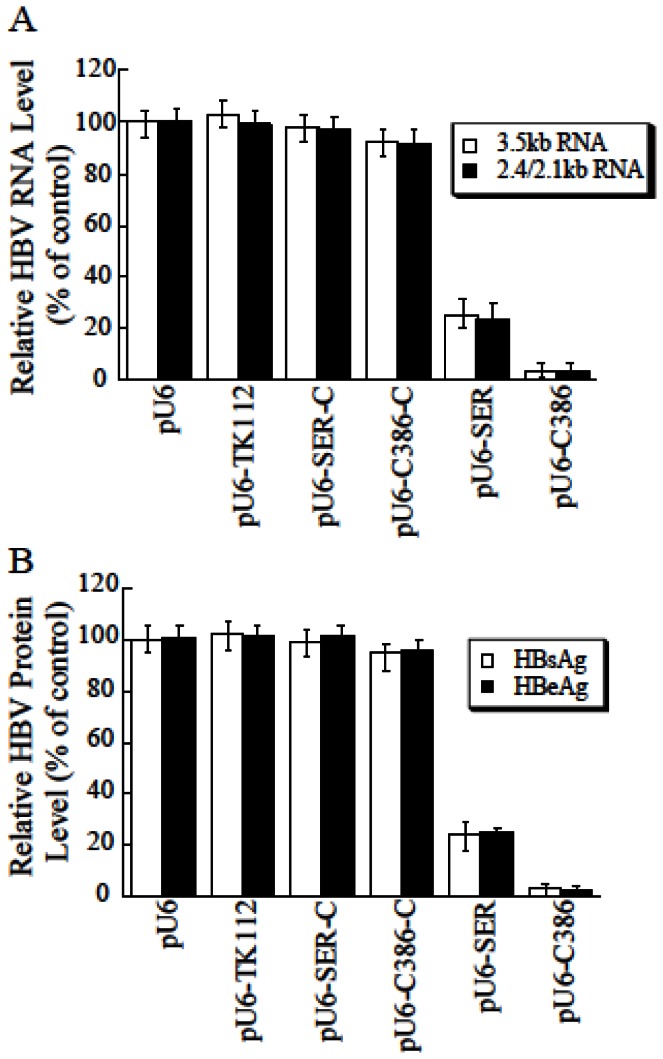
Inhibition of HBV RNA and protein expression in HepG2.2.15 cells treated with EGS-carrying *Salmonella* . At 48 hours post-treatment with *Salmonella* , RNA samples were isolated from cells, separated on denaturing gels, transferred to membranes, hybridized with a ^32^P-labeled HBV DNA probe, and analyzed with a phosphorimager, using Η1 RNA as the loading control (A). Cell culture media were also collected and the levels of HBsAg and HBeAg were determined by ELISA (B). The values, which are the means from triplicate experiments, represent the relative percentage of the levels of HBV RNAs, HBsAg, and HBeAg in cells treated with SL201 carrying different constructs, as compared to those in cells treated SL201 carrying empty vector pU6. The values in (A–B) are the means from triplicate experiments and the standard deviation is indicated by the error bars.

Since there is currently no cell culture system to support the full cycle of HBV infection effectively, we carried out experiments to study if *Salmonella* –mediated delivery of EGS affects HBV gene expression and viral production in cells that stably contained a replication-competent HBV DNA. HepG2.2.15 cells, which were derived from human hepatoma HepG2 cells and stably contained a transfected full length genome of HBV (ayw subtype), were used as the model system for HBV replication as these cells constitutively express hepatitis B surface (HBsAg) and e (HBeAg) antigens, and also support HBV genome replication [28]. We first treated these cells with SL201 carrying EGS constructs, and then isolated the *Salmonella* -containing cells by FACS analysis based on GFP expression and analyzed. Northern blot analysis was used to assay the expression of HBV transcripts of 3.5 kb and 2.4/2.1 kb, which represent the pre-C mRNA/pregenomic RNA and pre-S/L mRNA, respectively [15]. We used the level of actin mRNA, the expression of which is not regulated by HBV under the assay conditions [15], as the internal and loading control for the quantification of the expression of HBV transcripts (Figure 5). At 48 hours post-treatment, we observed a reduction of about 97% and 75% in the level of HBV transcripts in cells treated with SL201 carrying pU6-S-C386 and pU6-S-SER, respectively, while a reduction of less than 10% was observed in cells with SL201 containing pU6-S-C386-C, pU6-S-SER-C, or pU6-TK112 (Figure 5, lanes 1-4; Figure 6A). Consistent with observations in previous studies [9–11,32], no specific products of the cleavage of the target mRNAs by RNase P were detected in either Northern blot analyses or 5’ rapid amplification of cDNA ends (RACE) PCR assays between the RNA samples from cultured cells treated with SL201 carrying pU6-S-C386 and those with SL201 carrying pU6-S-C386-C or pU6-TK112.

It is anticipated that an inhibition of the levels of HBV transcripts would result in a reduction of HBV protein expression and production. We determined the levels of HBV production by assaying the expression of HBV antigens HBsAg and HBeAg in the supernatants of the cell cultures with ELISA [15]. We observed a reduction of about 97-98% and 75% in the level of HBsAg and HBeAg in cells treated with SL201 carrying pU6-S-C386 and pU6-S-SER, respectively (Figure 6B). A low level of inhibition (~8%) was found in cells treated with SL201 carrying pU6-S-C386-C or pU6-S-SER-C (Figure 6B), presumably due to an antisense effect because S-SER-C and S-C386-C exhibited similar binding affinity to the target sequence as S-SER and S-C386, respectively, but were unable to induce RNase P-mediated cleavage.

**Figure 7:**
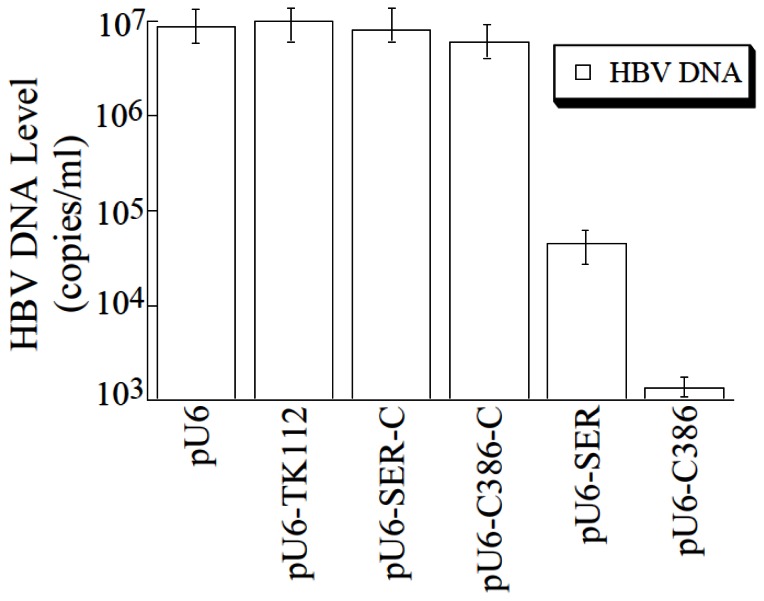
Inhibition of HBV DNA replication in HepG2.2.15 cells treated with EGS-carrying *Salmonella* . At 72 hours post-treatment, HBV capsid-associated DNAs were isolated from HepG2.2.15 cells that were treated with *Salmonella* carrying different EGS constructs. Levels of capsid-associated viral DNA were determined by qPCR. The values are the means from triplicate experiments and the standard deviation is indicated by the error bars.

*Salmonella* -mediated gene delivery of anti-HBV EGS also effectively inhibited HBV DNA replication. At 4 days post-treatment, a reduction of about 6,000- and 130-fold in the levels of intracellular capsid-associated HBV DNA, as measured by quantitative PCR (qPCR), was observed in cells treated with *Salmonella* carrying pU6-S-386 and pU6-S-SER, respectively, while a reduction of less than 10% was found in cells treated with SL201 containing pU6-S-C386-C, pU6-S-SER-C, or pU6-TK112 (Figure 7). 

## Discussion

The EGS-based technology represents an attractive approach for gene inactivation since it utilizes endogenous RNase P to generate highly efficient and specific cleavage of the target RNA [3,40]. However, little is known about the rate-limiting step of the EGS-targeting approach in cultured cells. Equally unclear is whether the efficacy of the EGSs can be improved, and if so, how it can be improved. In this study, we constructed EGSs that target an accessible region of HBV S mRNA. Our results indicated that an EGS variant, S-C386, is about 50 times more active [V_max(apparent)_ /K_m(apparent)_] in directing RNase P to cleave the S mRNA sequence *in vitro* than S-SER, an EGS derived from the natural tRNA^Ser^ sequence. Moreover, S-C386 inhibited HBV gene expression (i.e. HBV transcripts and HBsAg/HBeAg) in cultured cells by about 97-98% and was more effective than S-SER, which reduced HBV gene expression by about 75%. In contrast, a reduction of less than 10% in HBV gene expression and DNA replication was observed in cells that expressed control EGS S-C386-C, S-SER-C, or TK112. S-C386-C and S-SER-C exhibited similar binding affinity to s38 as S-C386 and S-SER, respectively, but were inactive in directing RNase P-mediated cleavage due to the presence of the mutations at the T-loop that precluded RNase P recognition (Figures 1 and 2 Table 1). Our results suggest that the observed reduction in viral gene expression and DNA replication with S-C386 and S-SER is primarily attributed to the specific targeted RNase P-mediated cleavage induced by these two EGSs as opposed to the antisense effect or other nonspecific effects of the EGSs. Moreover, our results suggest that the EGS (i.e. S-C386) that is more active [V_max(apparent)_ /K_m(apparent)_] in inducing RNase P to cleave the S mRNA sequence *in vitro* is also more effective in inhibiting HBV gene expression and DNA replication in cultured cells and that increasing the activity of EGS in directing RNase P cleavage *in vitro* may lead to improved efficacy in inhibiting gene expression in cultured cells.

Our results also suggest that the increased targeting activity of EGS S-C386 may be due to the enhanced stability of the mRNA-EGS complexes formed between S-C386 and the target HBV RNA sequence. S-C386 bound to substrate s38 with at least 70-fold greater affinity than S-SER (Table 1). Tertiary interactions between variable region and D-loop have been shown to be important for maintaining the tRNA conformation and RNase P cleavage [3,41]. In the s38-S-C386 complex, the 3’ region of s38 can be considered equivalent to the D-loop in a tRNA (Figure 1A, B, and E). It is reasonable to suggest that the additional interactions between s38 and S-C386 stabilize the mRNA-EGS complex and result in an enhanced binding affinity and increased targeting activity of the EGS. Thus, our study may provide a direction for the engineering and generation of highly active and effective EGS molecules by carrying out selection procedures and manipulation of the EGS domain to interact with the target RNA substrates. In vitro selection procedures have been widely used to generate highly active and functional RNA molecules (e.g. ribozymes and aptamers) that have increased activity [42–44]. Moreover, these procedures have been used to generate EGS molecules that direct human RNase P to cleave the mRNA encoding chloramphenicol acetyltransferase (CAT) [33]. Further studies of engineered EGSs as well as those studies on how to construct new *Salmonella* strains with better gene delivery activity, should facilitate the development of the EGS-based technology as a promising gene targeting approach for *in vivo* applications.

For EGSs to be successful as a therapeutic tool for blocking HBV replication, one of the most important issues is targeted delivery of these agents to specific types of cells and tissues such as hepatocytes in the liver. Several lines of evidence in our study indicate that EGS RNAs expressed following the *Salmonella* -mediated gene delivery are active and block HBV replication in cultured cells. First, targeted gene transfer of the EGS constructs by *Salmonella* yields substantial expression of the EGSs (Figure 4). Second, the presence of EGS sequences in *Salmonella* did not significantly affect the viability and gene transfer ability of the bacteria (Figure 3). Third, the EGS appeared to be active in directing RNase P-mediated cleavage in cultured cells. Reduced HBV gene expression and viral DNA replication were observed in cultured cells that were inoculated with SL201 carrying pU6-S-C386 and pU6-S-SER but not control constructs pU6-S-C386-C, pU6-S-SER-C, or pU6-TK112. Fourth, the extent of the reduction in the levels of HBV transcripts in cultured cells treated with *Salmonella* carrying the sequence of EGS S-C386 and S-SER correlated well with that in the levels of HBsAg/HBeAg and HBV DNA (Figures 5-7). Thus, the antiviral effect associated with the expression of functional EGS S-C386 and S-SER appeared to be due to the reduction of the levels of HBV transcripts, as a result of RNase P-mediated cleavage of the target S RNA directed by S-C386 and S-SER, respectively. These results suggest that following *Salmonella* -mediated delivery, EGSs effectively induce RNase P-mediated cleavage of its target RNA, resulting in a decrease of HBV gene expression and DNA replication and leading to an inhibition of viral infection and growth.

Our results also suggest that *Salmonella* -based vectors are effective in delivering EGS for gene-targeting applications in cultured cells. As a gene delivery tool, *Salmonella* -based vectors exhibit several unique and attractive features. For example, *Salmonella* -based vectors are low cost and easy to prepare. Another inportant aspect associated with *Salmonella* vector is the oral route administration of these bacteria [45,46]. Thus, *Salmonella* represents an attractive and promising gene delivery tool for gene therapy of human diseases, including those caused by HBV.

Compared to RNAi, the EGS-based technology is unique in inducing RNase P to cleave a target mRNA. EGSs have been previously shown to induce RNase P-mediated cleavage of HBV mRNAs in vitro and more recently, been shown to inhibit HBV gene expression and replication in cultured cells and in mice [20,21]. Our results presented here suggest that the level of inhibition of HBV gene expression and replication induced by the EGS-based technology in cultured cells appeared to be comparable with some of the best RNAi-mediated anti-HBV effects that were previously reported [17–19]. Our current study only focuses on investigating the activity of the constructed EGSs in vitro and in cultured cells. Further studies will be carried out to analyze the activity of the EGSs in inhibiting HBV gene expression and replication in animal models in vivo.

Hepatitis B virus (HBV) is a major human pathogen chronically infecting more than 400 million individuals worldwide [15]. Hepatocytes represent the major reservoir for HBV and this virus can establish both primary and chronic infections in these cells, leading to life-threatening complications such as cirrhosis, liver failure, and hepatocellular carcinoma [15,47]. Eliminating infection in hepatocytes is central to the treatment of HBV-associated diseases. Our study provides direct evidence that *Salmonella* -mediated gene transfer of EGS can effectively block HBV infection and replication in human cells. To further evaluate their anti-HBV activity, we can deliver and express the EGSs in hepatocytes and in the liver by oral administration of *Salmonella* vectors carrying the EGS constructs. Successful inhibition of HBV chronic infection requires treatment over a lengthy period. Further experiments may be needed to determine if *Salmonella-*mediated gene transfer can be long-lasting in hepatocytes, although it has been reported that transgene expression mediated by *Salmonella* vectors was detectable *in vivo* up to 1 month [48,49]. Moreover, repeated administration of the *Salmonella* vector to a patient with HBV chronic infection may be necessary and it is important to determine if immune response to the vector may arise and reduce the efficiency of the delivery.

Today, there is no definitive cure for chronic HBV infection. The current FDA-approved anti-HBV medications for the management of chronic HBV infections include alpha interferon, and five nucleoside analogues (lamivudine, adefovir, entecavir, telbivudine, and tenofovir) that act as potent inhibitors of viral polymerase but rarely cure HBV infection [15,47]. The major limitation of the current treatments is believed to be their inability to eliminate the preexisting and/or formation of HBV covalently closed circular DNA (cccDNA), which is essential for HBV replication and is responsible for the establishment of viral infection and persistence [50]. The EGS-based technology targets the mRNAs expressed from the cccDNA and presumably will not affect the level of HBV cccDNA. It will be interesting to evaluate the effect of EGSs in combination with anti-HBV cccDNA compounds [51] on inhibiting and eliminating chronic HBV infection. These studies will demonstrate the potential utility of EGSs for anti-HBV therapy.
